# Supplementary material for: Apoptosis inhibition reprograms alveolar myofibroblasts toward ductal myofibroblasts
Source: bioRxiv. 2025 May 28:2025.05.26.654588. Preprint. [Version 1] doi: 10.1101/2025.05.26.654588 (PMC12148055; doi:10.1101/2025.05.26.654588)
Supplement: Supplement 4 [file NIHPP2025.05.26.654588v1-supplement-4.pdf]

bioRxiv preprint doi: https://doi.org/10.1101/2025.05.26.654588; this version posted May 28, 2025. The copyright holder for this preprint (which was not certified by peer review) is the author/funder, who has granted bioRxiv a license to display the preprint in perpetuity. It is made available under aCC-BY 4.0 International license.

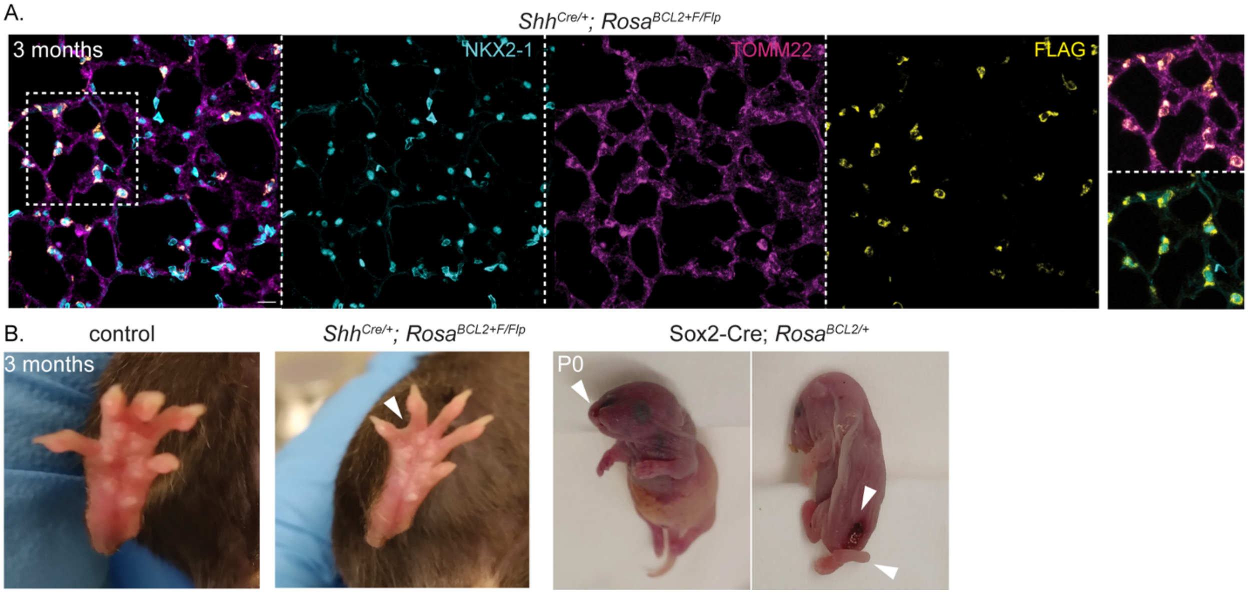

Supplemental Fig. 1. Mitochondrial localization and function of overexpressed BCL2.

A. Immunostaining of adult lungs shows that exogenous FLAG-tagged BCL2 activated by *Shh*^{Cre} is specific to epithelial cells (NKX2-1+) and colocalizes with a mitochondrial marker TOMM22. Scale bar: 20 um.

B. Webbing between digits four and five (arrowhead) from exogenous BCL2 activated by *Shh*^{Cre}.

C. Developmental defects including cleft palate (left), sacral spina bifida, and curly tail (right) from whole-embryo exogenous BCL2 activated by Sox2-Cre.

bioRxiv preprint doi: https://doi.org/10.1101/2025.05.26.654588; this version posted May 28, 2025. The copyright holder for this preprint (which was not certified by peer review) is the author/funder, who has granted bioRxiv a license to display the preprint in perpetuity. It is made available under aCC-BY 4.0 International license.

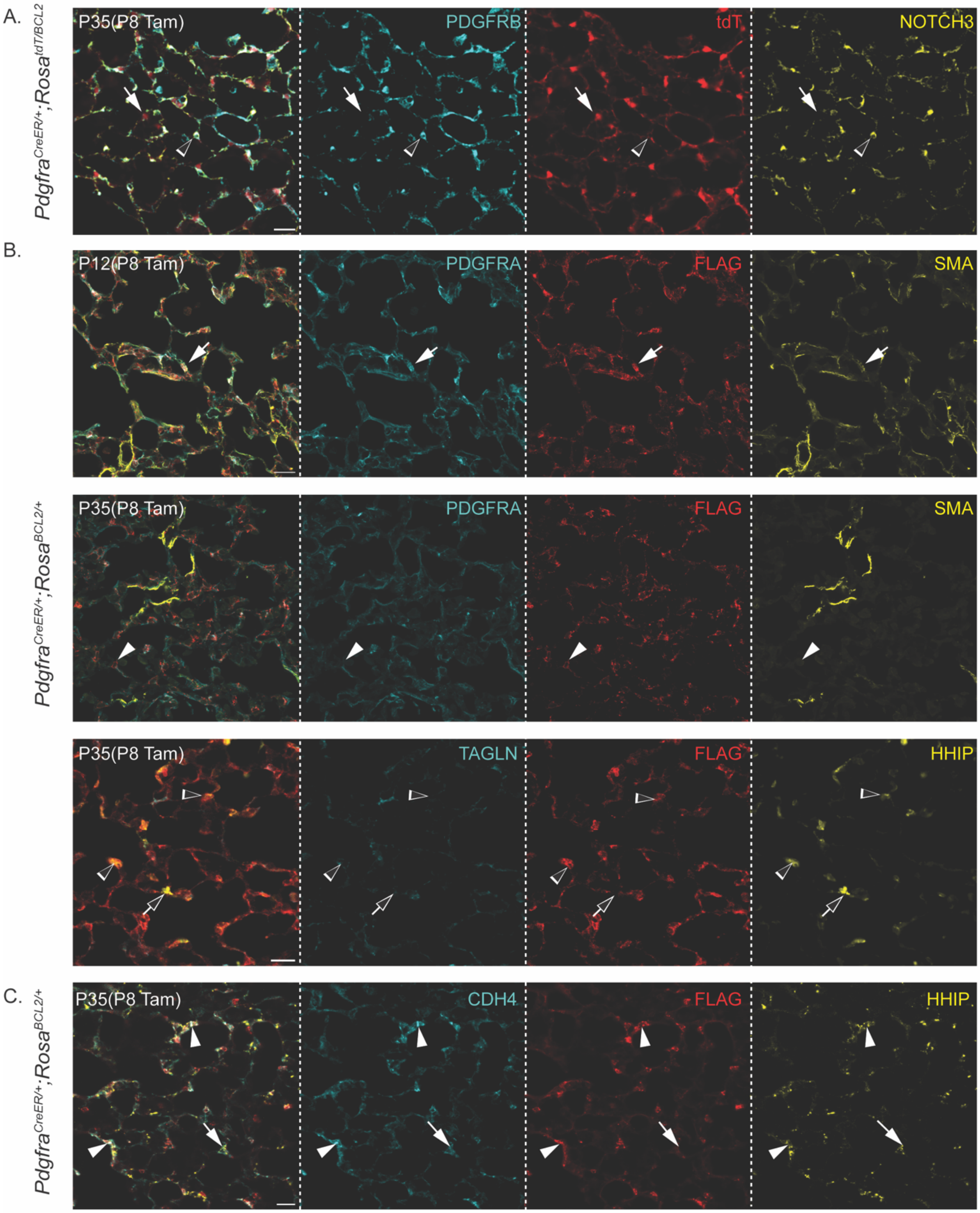

bioRxiv preprint doi: https://doi.org/10.1101/2025.05.26.654588; this version posted May 28, 2025. The copyright holder for this preprint (which was not certified by peer review) is the author/funder, who has granted bioRxiv a license to display the preprint in perpetuity. It is made available under aCC-BY 4.0 International license.

Supplemental Fig. 2. Persistent AMFs remain in the epithelial axis and downregulate contractile proteins.

A. Immunostaining of mature lungs, representative of at least 4 mice (same below), shows that persistent cells (arrow) do not express PDGFRB or NOTCH3, markers of the vascular axis (open arrowhead).

B. Immunostaining of neonatal and mature BCL2 lungs shows that neonatal AMFs (FLAG+PDGFRA^{high}; arrow in top row) are ACTA2+, while persistent AMFs (FLAG+; arrowhead in middle row) downregulate PDGFRA and ACTA2. Bottom row: DMF-like cells (FLAG+HHIP+; open arrowhead) and DMF (FLAG-HHIP+; open arrow) cells also downregulate TAGLN.

C. Immunostaining of mature BCL2 lungs shows that DMF-like cells (FLAG+; arrowhead) co-express HHIP and CDH4, markers of DMFs (FLAG-; arrow). All scale bars: 20 um.

bioRxiv preprint doi: https://doi.org/10.1101/2025.05.26.654588; this version posted May 28, 2025. The copyright holder for this preprint (which was not certified by peer review) is the author/funder, who has granted bioRxiv a license to display the preprint in perpetuity. It is made available under aCC-BY 4.0 International license.

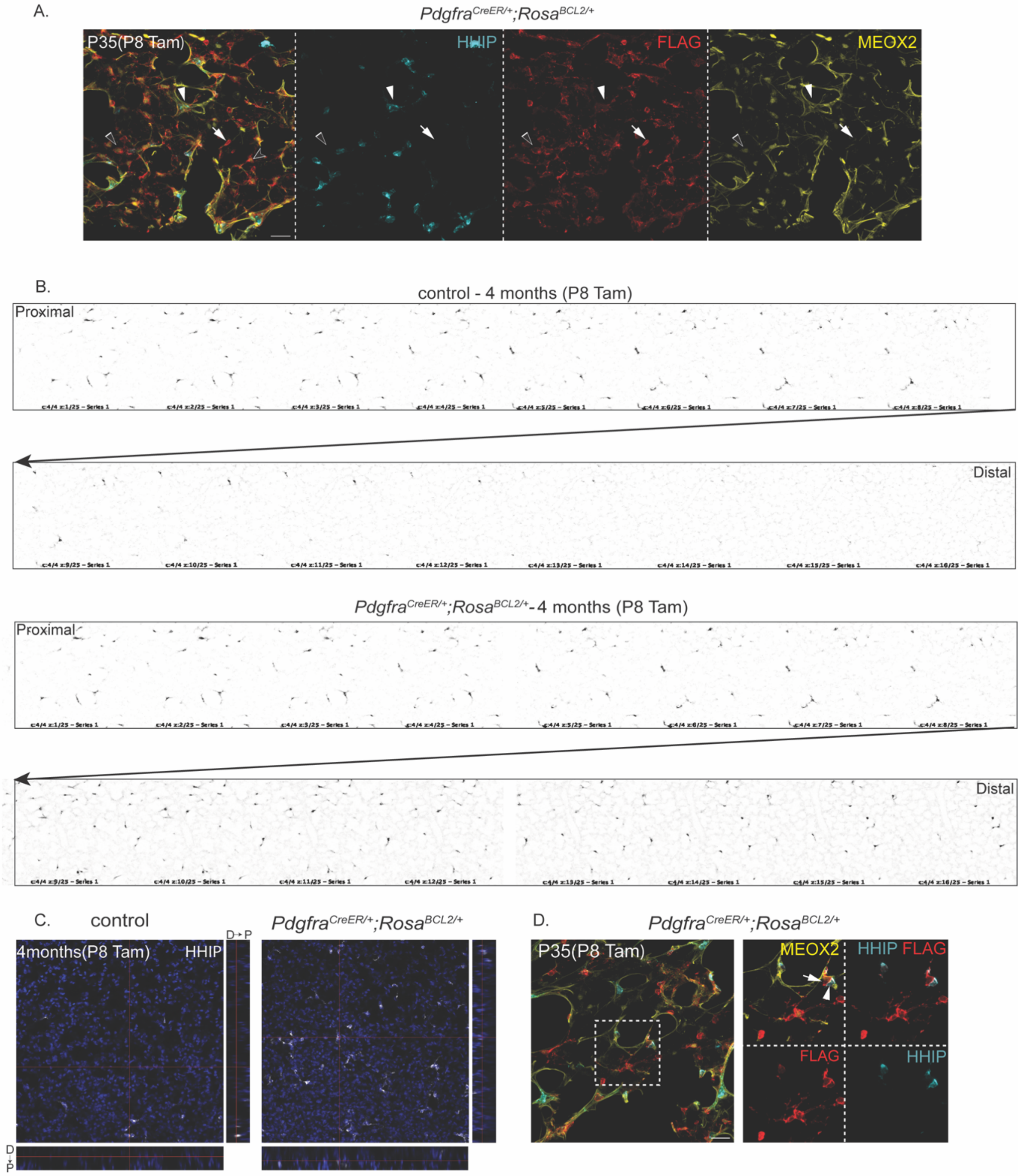

bioRxiv preprint doi: https://doi.org/10.1101/2025.05.26.654588; this version posted May 28, 2025. The copyright holder for this preprint (which was not certified by peer review) is the author/funder, who has granted bioRxiv a license to display the preprint in perpetuity. It is made available under aCC-BY 4.0 International license.

Supplemental Fig. 3. DMF-like cells surround distal alveolar ducts while SCMF-like cells surround alveoli.

A. Immunostaining of mature BCL2 lung shows DMF-like cells (FLAG+HHIP+; arrowhead) surrounding distal alveolar ducts, while SCMF-like cells (FLAG+HHIP-; arrow) reside in the terminal alveoli. Like DMFs, DMF-like cells are associated with thick elastin fibers (autofluorescence in MEOX2 staining), whereas SCMF-like cells are associated with none or thin elastic fibers. Open arrowhead: FLAG+MEOX2+ AF1s targeted by *Pdgfra*^{CreER}.

B. Single-section montages of Z stacks of wholemount immunostained lung strips shows that DMFs (HHIP+) in the control are restricted to proximal alveolar ducts, whereas DMF-like cells (HHIP+) in the BCL2 lung expand distally.

C. Orthogonal views of Z stacks of wholemount immunostained lung strips show more HHIP+ cells located distally in the BCL2 lung than the control. D: distal; P: proximal.

D. Immunostaining of mature BCL2 lungs showing seemingly juxtaposition of persistent SCMF-like (arrow) and DMF-like (arrowhead) cells due to the 3D tissue complexity. All scale bars: 20 um.

bioRxiv preprint doi: https://doi.org/10.1101/2025.05.26.654588; this version posted May 28, 2025. The copyright holder for this preprint (which was not certified by peer review) is the author/funder, who has granted bioRxiv a license to display the preprint in perpetuity. It is made available under aCC-BY 4.0 International license.

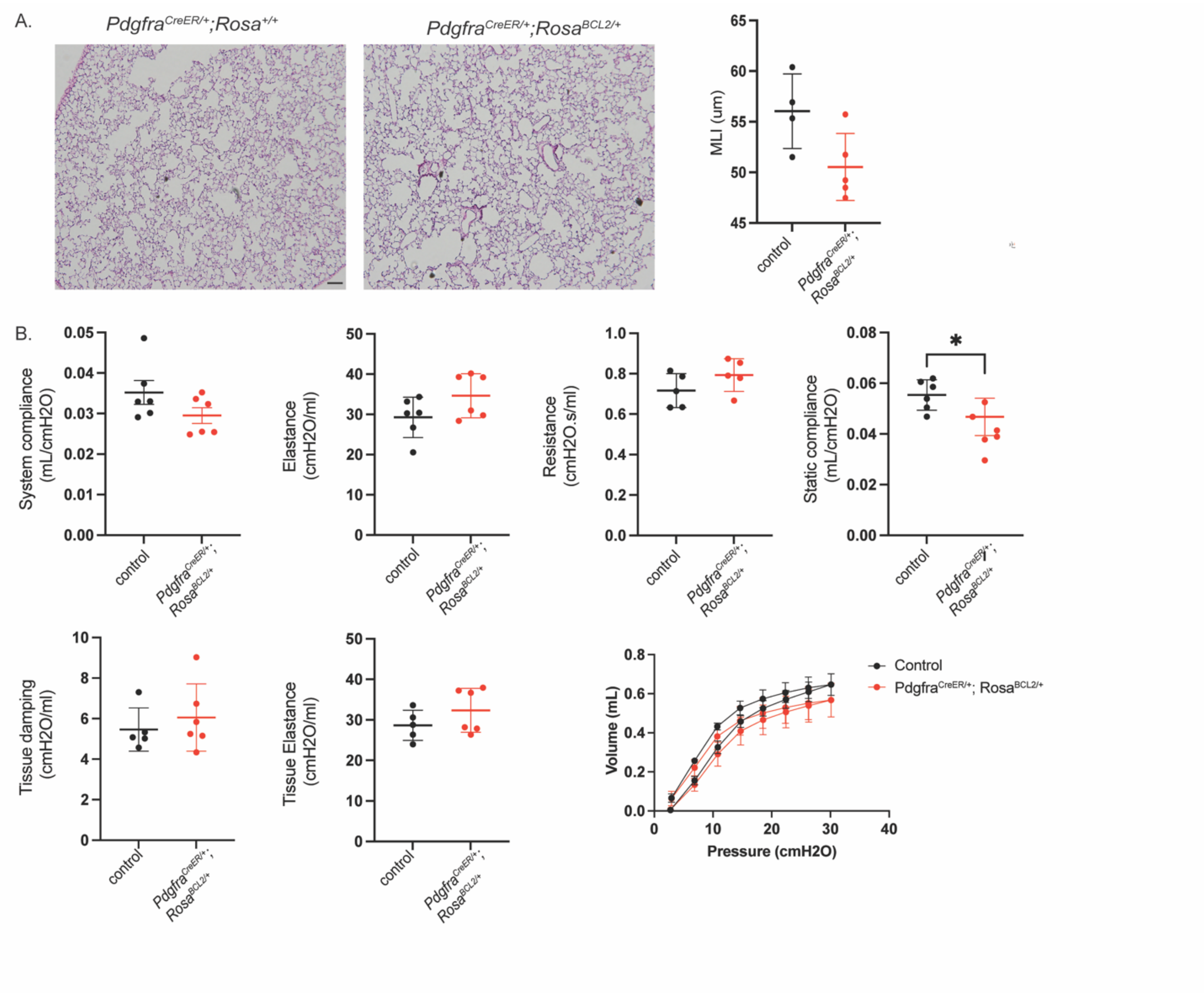

Supplemental Fig. 4. Persistent AMFs minimally affect lung morphology or mechanics.

A. H&E staining of BCL2 and littermate control lungs and mean linear intercept (MLI) quantification show no significant difference (n=4; 3 images/mouse). Scale bar: 20 um.

B. FlexiVent measurements of lung mechanics show minimal change in BCL2 lungs except for a small decrease in static compliance. See Table S1 for raw data.

bioRxiv preprint doi: https://doi.org/10.1101/2025.05.26.654588; this version posted May 28, 2025. The copyright holder for this preprint (which was not certified by peer review) is the author/funder, who has granted bioRxiv a license to display the preprint in perpetuity. It is made available under aCC-BY 4.0 International license.

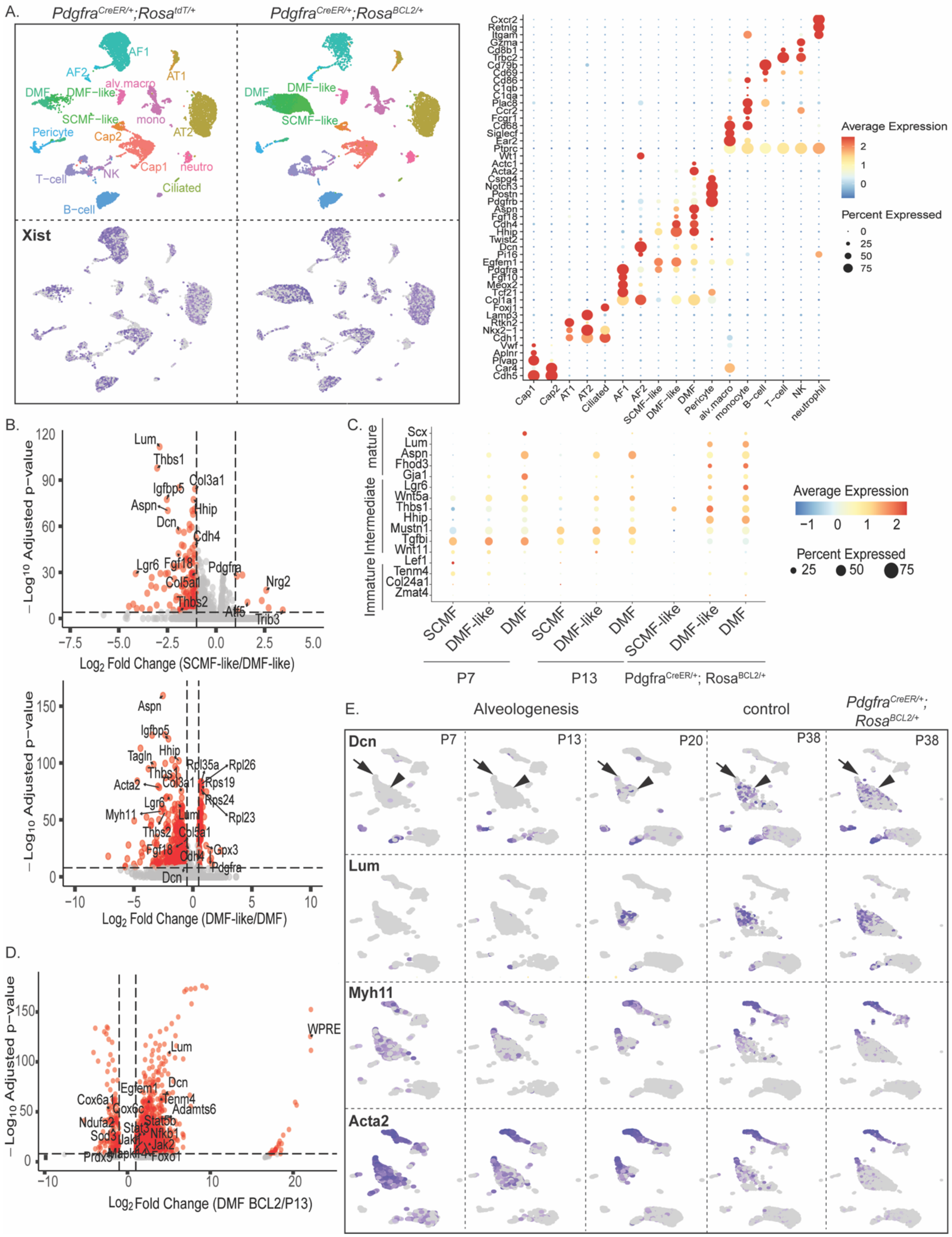

bioRxiv preprint doi: https://doi.org/10.1101/2025.05.26.654588; this version posted May 28, 2025. The copyright holder for this preprint (which was not certified by peer review) is the author/funder, who has granted bioRxiv a license to display the preprint in perpetuity. It is made available under aCC-BY 4.0 International license.

Supplemental 5. Persistent AMFs change from contractile to non-contractile fibroblasts.

A. UMAP and dot plot of mature lungs color-coded by cell types of epithelial, endothelial, mesenchymal, and immune lineages (top) and feature plots of Xist showing comparable results from male and female lungs (bottom).

B. Volcano plots comparison of SCMF-like and DMF-like (Top), and DMF-like and DMFs (Bottom) from mature BCL2 lungs, showing upregulation of genes associated with stages of myofibroblasts maturation. Genes such as *Pdgfra* and ribosomal genes (*Rpl28*, *Rpl23*, *Rps19*) found in SCMF-like and DMF-like represent immature and intermediate DMFs, while upregulation of DMF markers such as *Fgf18*, *Thbs2*, *Myh11*, and *Lum* corresponds to more mature DMFs.

C. Dot plots show that previously reported immature (*Zmat4*, *Col24a1*,*Tenm4*, *Lef1*) intermediate (*Mustn1*, *Hhip*, *Thbs1*, *Wnt5a*), and mature (*Scx*, *Lum*, *Aspn*, *Fhod3*) myofibroblast markers of the developing lungs (P7 and P13) generally align with SCMF-like cells, DMF-like cells, and DMFs in BCL2 lungs.

D. Volcano plot comparison of DMFs from P13 control and mature BCL2 lungs showing upregulation of mature DMF genes (*Lum* and *Dcn*) and genes associated with survival and differentiation (*Jak1*, *Jak2*, *Stat3*) in BCL2 lungs. Upregulation of the lineage marker WPRE suggests that some persistent cells are reprogrammed completely into DMFs.

E. Feature plots showing that like DMFs (arrow), DMF-like cells (arrowhead) mature by upregulating matrix genes Dcn and Lum and downregulating contractile genes Myh11 and Acta2.

bioRxiv preprint doi: https://doi.org/10.1101/2025.05.26.654588; this version posted May 28, 2025. The copyright holder for this preprint (which was not certified by peer review) is the author/funder, who has granted bioRxiv a license to display the preprint in perpetuity. It is made available under aCC-BY 4.0 International license.

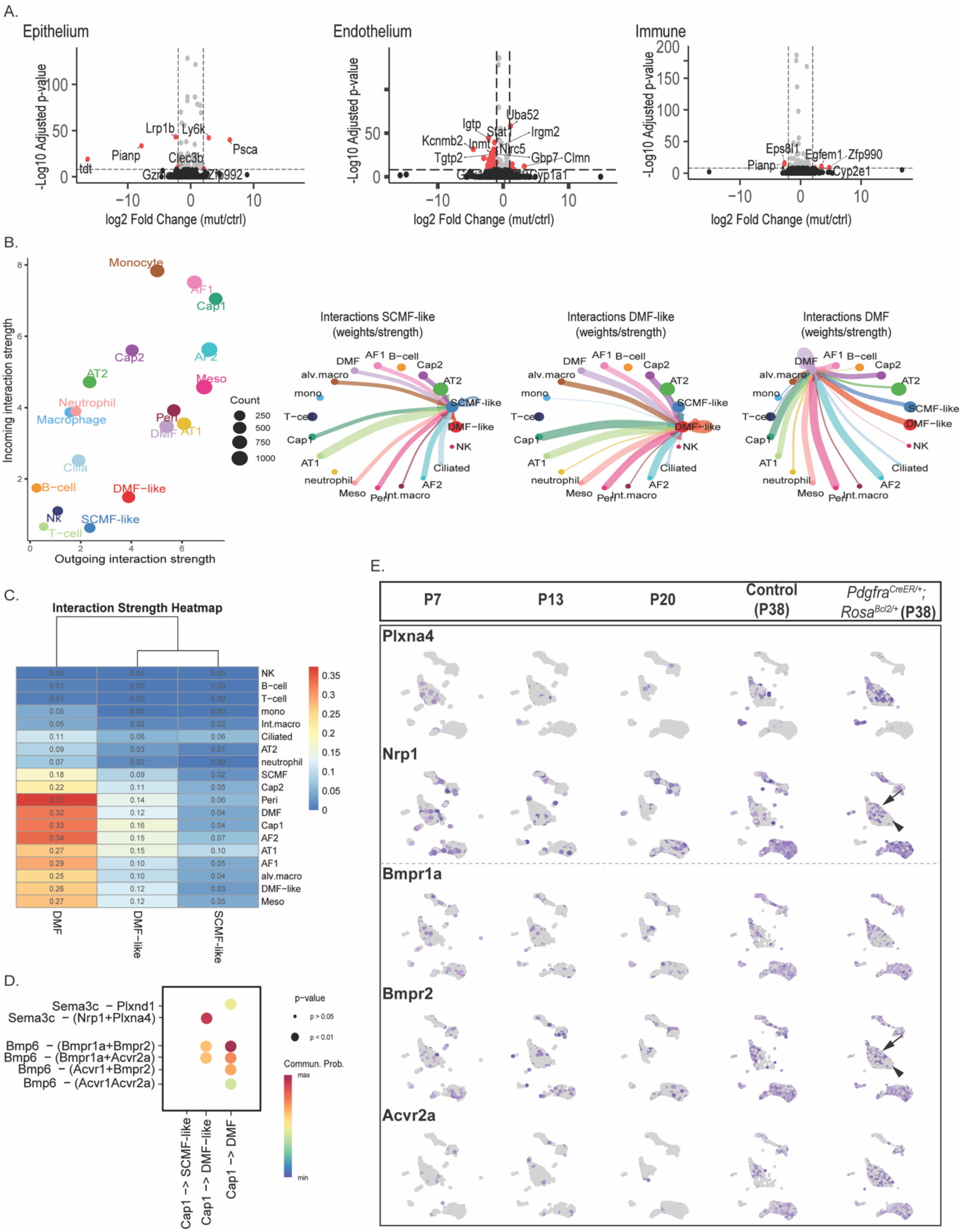

bioRxiv preprint doi: https://doi.org/10.1101/2025.05.26.654588; this version posted May 28, 2025. The copyright holder for this preprint (which was not certified by peer review) is the author/funder, who has granted bioRxiv a license to display the preprint in perpetuity. It is made available under aCC-BY 4.0 International license.

Supplemental 6. Limited interactions between persistent AMFs and other cell lineages

A. Volcano plots comparison of epithelial, endothelial and immune lineage cells between P38 *Pdgfra*$^{CreER/+}$; *Rosa*$^{BCL2/+}$ and control lungs showing minimal changes in non-mesenchymal cell lineages.

B. Left: CellChat ligand-receptor analysis of BCL2 lungs showing low outgoing and incoming interaction strength of SCMF-like and DMF-like cells. Right: Circle plots showing increasing interaction weights/strength for SCMF-like cells, DMF-like cells, and DMFs.

C. Heatmap of interaction strength showing strongest interactions between DMFs and CAP1s, DMFs, pericytes, and AF2s.

D. Differential incoming ligand-receptor interactions (Semaphorin and Bmp) of DMF-like cells, but not SCMF-like cells, with CAP1s, possibly mediating DMF-like reprogramming.

E. Feature plots of neonatal and mature lungs show that expression of potential Semaphorin (Nrp1) and Bmp (Bmpr2) receptors mediating DMF-like reprogramming is higher in DMF-like cells (arrow) than SCMF-like cells (arrowhead). Plxna4, a receptor for Sema3c/3d, is specific for mesenchymal cells of the epithelial axis.

bioRxiv preprint doi: https://doi.org/10.1101/2025.05.26.654588; this version posted May 28, 2025. The copyright holder for this preprint (which was not certified by peer review) is the author/funder, who has granted bioRxiv a license to display the preprint in perpetuity. It is made available under aCC-BY 4.0 International license.

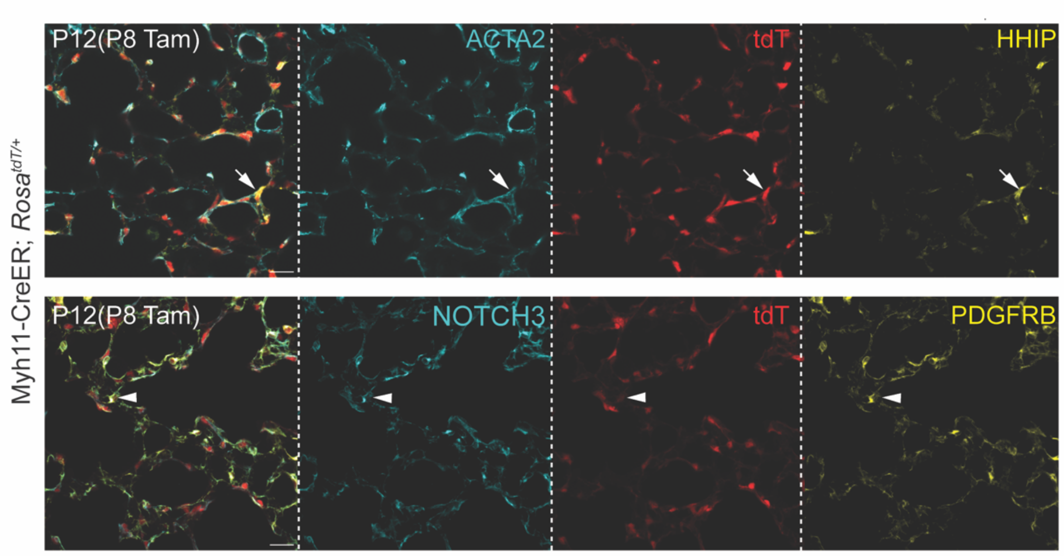

Supplemental 7. Additional characterization of the Myh11-CreER driver

Immunostaining of acutely labeled lungs, representative of at least 4 mice, showing that Myh11-creER targets DMFs (HHIP+; arrow) and pericytes (PDGFRB+NOTCH3+; arrowhead), although pericyte tdT is lower. Scale bar: 20 um.
